# Mefloquine Inhibits Esophageal Squamous Cell Carcinoma Tumor Growth by Inducing Mitochondrial Autophagy

**DOI:** 10.3389/fonc.2020.01217

**Published:** 2020-07-28

**Authors:** Yifei Xie, Jing Zhang, Bingbing Lu, Zhuo Bao, Jimin Zhao, Xianyu Lu, Yaxing Wei, Ke Yao, Yanan Jiang, Qiang Yuan, Xiaofan Zhang, Bo Li, Xinhuan Chen, Zigang Dong, Kangdong Liu

**Affiliations:** ^1^Department of Pathophysiology, School of Basic Medical Sciences, AMS, Zhengzhou University, Zhengzhou, China; ^2^Henan Provincial Cooperative Innovation Center for Cancer Chemoprevention, Zhengzhou, China; ^3^China-US (Henan) Hormel Cancer Institute, Zhengzhou, China; ^4^State Key Laboratory of Esophageal Cancer Prevention and Treatment, Zhengzhou, China; ^5^Cancer Chemoprevention International Collaboration Laboratory, Zhengzhou, China

**Keywords:** esophageal squamous cell carcinoma (ESCC), mefloquine (MQ), mitochondria, autophagy, succinate dehydrogenase complex subunit C (SDHC)

## Abstract

Esophageal squamous cell carcinoma (ESCC) has a worldwide impact on human health, due to its high incidence and mortality. Therefore, identifying compounds to increase patients' survival rate is urgently needed. Mefloquine (MQ) is an FDA-approved anti-malarial drug, which has been reported to inhibit cellular proliferation in several cancers. However, the anti-tumor activities of the drug have not yet been completely defined. In this study, mass spectrometry was employed to profile proteome changes in ESCC cells after MQ treatment. Sub-cellular localization and gene ontology term enrichment analysis suggested that MQ treatment mainly affect mitochondria. The KEGG pathway enrichment map of down-regulated pathways and Venn diagram indicated that all of the top five down regulated signaling pathways contain four key mitochondrial proteins (succinate dehydrogenase complex subunit C (SDHC), succinate dehydrogenase complex subunit D, mitochondrially encoded cytochrome c oxidase III and NADH: ubiquinone oxidoreductase subunit V3). Meanwhile, mitochondrial autophagy was observed in MQ-treated KYSE150 cells. More importantly, patient-derived xenograft mouse models of ESCC with SDHC high expression were more sensitive to MQ treatment than low SDHC-expressing xenografts. Taken together, mefloquine inhibits ESCC tumor growth by inducing mitochondrial autophagy and SDHC plays a vital role in MQ-induced anti-tumor effect on ESCC.

## Introduction

Esophageal squamous cell carcinoma (ESCC), which accounts for 90% of esophageal cancer, is the sixth leading cause of cancer-associated mortality, especially in several developing countries (1). Despite developments in medical and surgical treatment, the 5-year survival rate of ESCC is only 15–25%, largely because of recurrence of primary treatment and late stage diagnosis (2, 3). Currently, radiotherapy and chemotherapy are the main methods for ESCC treatment and recurrence prevention, but their side effects and limited effects make it an urgent task to identify new drugs with low toxicity and high efficiency. Screening the library of drugs already approved by the FDA to discover drugs for this purpose may be an effective shortcut.

Mitochondria, as the main oxidative energy producers in eukaryotic cells, consist of five protein complexes, including NADH dehydrogenase (Complex I), succinate dehydrogenase (Complex II), ubiquinol cytochrome c oxidoreductase (Complex III), cytochrome c oxidase (Complex IV), and ATP synthase (Complex V). Dysregulated protein levels of any of these complexes can be indicative of mitochondrial disorder (4). Furthermore, mitochondrial autophagy, a conserved self-digestion process, plays a key role in the maintenance of cell homeostasis, and dysregulation of autophagy often occurs during tumor development (5). Activation of autophagy not only helps cells to survive in stressful conditions (6), but can also induce cell death (7). Therefore, mitochondrial autophagy regulation has been a promising strategy for cancer treatment (8).

In previous reports, mefloquine (MQ), an anti-malarial drug, has been shown to inhibit cellular proliferation in several cancer cell lines, including colorectal, gastric, prostate, and breast cancers (9–11); however, the global changes in the proteome and the anti-tumor effect of MQ have not been fully elucidated. In this study, we probed that protein changes in ESCC cells after MQ treatment are involved in mitochondrial autophagy. MQ can strongly suppress ESCC tumor growth in patient-derived xenograft mouse models.

## Materials and Methods

### Chemicals and Reagents

MQ was purchased from TCI Chemicals (#M2313, Tokyo, Japan) and Med Chem Express (#HY-17437A, Monmouth, NJ, USA). The primary antibodies against SDHC (#ab155999), SDHD (#ab189945), MTCO3 (# ab110259), NDUFV3 (# ab200227), and OXPHOS (#ab110413) were purchased from Abcam (Cambridge, UK). The primary antibody against LC3- A/B (#12741) was purchased from Cell Signaling Technology (Danvers, MA, USA). Hoechst 33342 (#23491-52-3) was purchased from Solarbio Life Sciences (Beijing, China). Reactive oxygen species (ROS) assay kit (S0033S) and NAD+/NADH assay kit (S0175) was obtained from Beyotime (Shanghai, China). ATPlite (Luminescence ATP detection) assay kit (6,016,943) was from PerkinElmer (Waltham, MA). N-acridine orange was purchased from Thermo Fisher scientific (Catalog number # A1372, Waltham, MA).

### Animals and Diets

CB17/SCID mice (5–6 weeks) were purchased from Vital River (Beijing, China). Mice were housed in a pathogen-free environment designed for immunodeficient mice under conditions of 20 ± 2°C, 50 ± 10% relative humidity, 12-h light/dark cycles. They were provided with food and water *ad libitum*. All procedures involving animals in this study were approved by the Research Ethics Committee of Zhengzhou University.

### Cell Culture

Shantou human embryonic esophageal (SHEE) cells were obtained from Shantou University (12, 13). ESCC cell lines KYSE150 and KYSE450 cells were purchased from the Chinese Academy of Sciences cell bank (Shanghai, China). Before freezing and culture, cells were cytogenetically tested by STR-Promega and authenticated (August, 2014 and July, 2017) as described previously (14). Cells were cultured at 37°C in a 5% CO_2_ incubator.

### Cell Proliferation Assay

SHEE cells (8 × 10^3^/well), KYSE150 cells (3 × 10^3^/well) and KYSE450 cells (5 × 10^3^/well) were seeded into 96-well plates, and different concentrations of MQ (#HY-17437A, Med Chem Express, Monmouth, NJ, USA) (0, 1, 2.5, 5, and 10 μM) were added to cells. Plates were taken out at 0, 24, 48, 72, and 96 h. DAPI was used to stain the cells' nuclei, and then cells were counted by an In Cell Analyzer 6,000 (GE).

### Anchor Independent Cell Growth Experiment

KYSE150 and KYSE450 cells (8 × 10^3^/well) were suspended in Eagle's Basal Medium (BME) with 10% fetal bovine serum (FBS) and 0.33% agar in the top layer, and different doses of MQ (0, 1, 2.5, 5, and 10 μM) were added into the mixed agar both in the top layer and base layer. The cultures were maintained at 37°C in a 5% CO_2_ incubator for about 1 week, and then colonies were scanned and counted using an In Cell Analyzer 6,000 (GE).

For plate cloning assay, cells were seeded into 6-well plates (200 cells/well) and treated with different doses of MQ (0, 1, 2.5, 5, and 10 μM). After 8 days culture, clones were stained with 0.5% crystal violet for 4 min. Clones were then photographed and counted.

### Apoptosis Assay

KYSE150 and KYSE450 cells (9 × 10^4^/dish) were seeded into 60 mm dishes, after 16 h incubation, cells were treated with DMSO and MQ (10 μM) for 48 and 72 h. Cells were collected and washed 2 times with PBS, cells were then resuspended with 250 μL apoptosis buffer, incubated for 15 min at room temperature with annexin V and propidium iodide. Samples were then analyzed using a flow cytometer.

### Cell Sample Preparation and Proteomics Analysis

KYSE150 cells (1 × 10^6^) were treated with 10 μM MQ for 24 h, after which cells were collected for protein extraction. Samples were digested with trypsin, and then fractionated by high pH reverse-phase HPLC using Agilent 300 Extend C18 column (5 μm particles, 4.6 mm, 250 mm length). Briefly, peptides were first separated with a gradient of 8% to 32% acetonitrile (pH 9.0) over 60 min into 60 fractions. Then, peptides were combined into 6 fractions and dried by vacuum centrifuging. The peptides were subjected to NSI source followed by tandem mass spectrometry (MS/MS) in Q ExactiveTM Plus (Thermo) coupled online to the UPLC. Data were obtained by searching the database for identified peptides which were assembled as proteins. The MS/MS data were processed using the Maxquant search engine (v.1.5.2.8) and then analyzed.

### Reactive Oxygen Species (ROS) Assay

KYSE150 and KYSE450 cells (9 × 10^4^/dish) were seeded into 100 mm dishes, after 16 h incubation, cells were treated with DMSO and MQ (10 μM) for 24 h. DCFH-DA was diluted into fresh medium (no FBS), the final concentration was 10 mM. Then, removed the medium and washed the cells 2 times with PBS, 1.5 ml of diluted DCFH-DA (10 μM) was added into the dishes. Cells were incubated for 20 min in 37°C incubator and mixed upside down every 3–5 min. After washed 3 times with fresh medium (no FBS), ROS levels in cells were measured with flow cytometer.

### NAD^+^/NADH Assay

KYSE150 and KYSE450 cells (1.8 × 10^5^/dish) were seeded into 60 mm dishes, after 16 h incubation, cells were treated with DMSO and MQ (10 μM) for 24 h. NAD^+^/NADH extract solution was added to cells after removing medium. Beating the cells lightly with NAD^+^/NADH extract solution to promote cell lysis. The mixture was centrifuged with 12,000 *g* for 10 min, 4°C, collected the supernatant, and 70 μL of supernatant was incubated at 60°C for 30 min to break down NAD^+^. Then, the NADH standard (0, 0.25, 0.5, 1, 2, 4, 6, 8, and 10 μM) (20 μL/well) was added into 96-well plates, as well as the supernatant sample, alcohol dehydrogenase (90 μL/well) was also added into 96-well plates. Color developing solution was added into the 96-well plates (10 μL/well), after incubation for 30 min, the absorbance was measured with microplate reader at 450 nm wavelength.

### ATPlite (Luminescence ATP Detection) Assay

KYSE150 and KYSE450 cells (4 × 10^3^/dish) were seeded into 96-well plate, after MQ treatment for 24 h, cells were lysed with mammalian lysis solution buffer (50 μl/well) for 5 min. Then substrate solution was added into wells, after 10 min incubation in dark, ATP generation was tested by measuring luciferase activity using a luminometer (Luminoskan Ascent, Thermo Electro, Waltham, MA).

### Transmission Electron Microscopy

In order to observe any morphological change of mitochondria in KYSE150 cells, both DMSO and MQ-treated (10 μM) KYSE150 cells were fixed in 2.5% glutaraldehyde for 24 h at 4°C, and post-fixed with 1% osmium tetroxide for 2 h. Dehydration was performed in an acetone gradient series and samples were then embedded in Spurr's resin. Semithin sections (700 nm) were analyzed with 1% toluidine blue and thin spinal cord sections (60–90 nm) were stained with aqueous 5% uranyl acetate, followed by 1% lead citrate. Material analysis and image capture were performed with transmission electron microscopy (150 kV, HT7700).

### Immunofluorescence Microscopy

KYSE150 cells (2 × 10^5^) were seeded onto slides in 12-well plates, and 10 μM MQ was added into culture medium for 24 h. Cells were fixed with 4% paraformaldehyde for 30 min at room temperature. 0.5% TritonX-100 was used for increasing antigen accessibility. After PBST wash, cells were blocked for non-specific antigen with 1% BSA/PBST for 1.5 h at room temperature. Cells were then incubated with primary antibodies (LC3 A/B, 1:50 dilution) overnight at 4°C. For the co-localization analysis of mitochondrial marker MTCO1 and autophagic marker LC3, we used the antibodies including MTCO1 and LC3 A/B (1:50 dilution). After being washed with 1% BSA/PBST, cells were stained with secondary antibody (Invitrogen) for another 1.5 h at room temperature, with light avoidance; 5 μg/ml DAPI was used to stain cell nuclei for 5 min at 37°C. With coverslips applied, cells were observed and photographed with GE Deltavision.

### N-Acridine Orange (NAO) Staining Analysis

KYSE150 and KYSE450 (1.8 × 10^5^) cells were seeded into 60 mm dishes, after 16 h, cells were treated with DMSO and MQ (10 μM) for 24 h. Then, cells were collected, and the concentration was adjusted to 1.5 × 10^6^/ml with fresh medium. NAO (5 μM) was added into the medium. The cells were incubated for 15 min at 20°C. The cells were then analyzed with flow cytometer.

### Plasmid Construction, Transfection, and Lenti-Virus Transduction

KYSE150 and KYSE450 cell lines were transfected with short hairpin RNA (sh*SDHC*), and the sh*SDHC* plasmids were cloned into the lentiviral expression vector plko.1. Human sh*SDHC* full hairpin sequence is #1. 5′ CCGGAGTACCTGGTAGACCATAATACTCGAGTATTATGGTCTACCAGGTACTTTTTTG3′; #2. 5′ CCGGATTGCCTCCGAGCCCACTTTACTCGAGTAAAGTGGGCTCGGAGGCAATTTTTTG3′. The transfection process was conducting according to protocol as described before (13). The transduction efficiency was examined by Western blot. Cell growth and clone formation ability of knock-down cells were compared with mock-transfected cells.

### Patient-Derived Xenograft (PDX) Mouse Model Establishment

This study was approved under a protocol approved by the Zhengzhou University Institutional Animal Care and Use Committee (Zhengzhou, Henan, China). ESCC tissues were obtained from the first Affiliated Hospital of Zhengzhou University, and written informed consent was provided by all patients for the use of the tissue samples. The procedure of PDX mouse model establishment has been described previously (15). About 1 week after tumor implantation, the mice were randomly divided into 3 groups with 10 mice per group: vehicle control (0.9% saline), low dose MQ (50 mg/kg) and high dose MQ (200 mg/kg) of MQ (#M2313, TCI Chemicals, Tokyo, Japan). Body weight was monitored three times per week, and tumor volume was measured twice a week. Tumor volume was calculated using the formula: V = LD x (SD)^2^/2, where V is the tumor volume, LD is the longest tumor diameter and SD is the shortest tumor diameter. When the average tumor volume of the control group reached 1,000 mm^3^, the mice were anesthetized, and the tumor masses were weighed and then immediately placed on ice. The tumor masses were divided into two parts. One part was fixed in formalin for hematoxylin and eosin (HE) and immunohistochemistry (IHC) analysis, and the second part was frozen at −80°C for protein detection. All research protocols were approved by the Research Ethics Committee of Zhengzhou University.

### Immunohistochemistry Analysis

Formalin-fixed tumor tissues were paraffin embedded and cut into 4 μm sections and placed onto slides. Tissue sections were dewaxed in xylene and hydrated in gradient alcohol (50, 75, 95, 100, and 100%), and washed 3 times with TBST; 10 mM citrate buffer (pH 6.0) was used to unmask the epitopes. This was followed by 0.03% hydrogen peroxide for 5 min at room temperature to block endogenous peroxidase activity. Tissues were incubated with SDHC primary antibody (1:50) at 4°C overnight. Tissues were then incubated with HRP-IgG secondary antibody at 37°C for 15 min. Samples were stained with DAB and counterstained with hematoxylin. After being dehydrated, slides were mounted and then scanned using Tissue Faxes (version 4.2), and positive cells were calculated using Image Pro Plus software program (Media Cybernetics, Rockville, MD).

### Statistical Analysis

One-way ANOVA or a non-parametric test was used for statistical analysis; *p* < 0.05 was considered statistically significant. All quantitative data are expressed as mean values ± S.D. as indicated.

## Results

### MQ Inhibits Proliferation and Anchor-Independent Cell Growth of ESCC Cells

**Figure 1:**
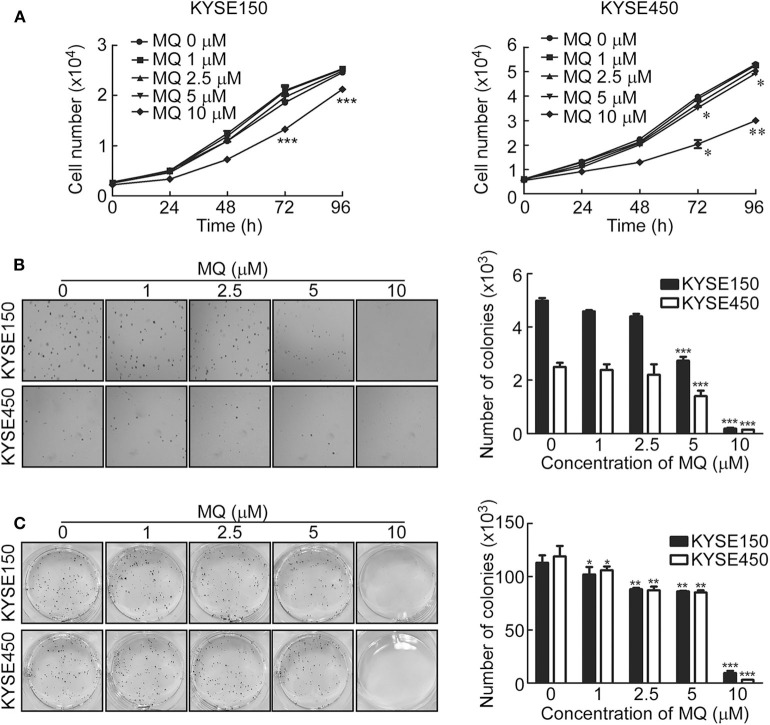
Mefloquine inhibits proliferation and anchorage- independent cell growth of KYSE150 and KYSE450 cells. **(A)** Cell count after treatment with varying doses of MQ (0, 1, 2.5, 5, 10 μM) in KYSE150 and KYSE450 cells after 0, 24, 48, 72 and 96 h. *p < 0.05, **p < 0.01, ***p < 0.001 (ANOVA); **(B)** Soft agar assay of KYSE150 and KYSE450 cells treated with different concentrations of MQ (0, 1, 2.5, 5 and 10 μM) for ~7 days. **p < 0.01, ***p < 0.001 (ANOVA); **(C)** Clone formation assay of KYSE150 and KYSE450 cells treated with different concentrations of MQ (0, 1, 2.5, 5 and 10 μM), stained with purple crystal after ~7 days. All data are shown as means ± S.D. The asterisks (*, **, ***) indicate a significant decrease (*p* < 0.05, *p* < 0.01, *p* < 0.001, respectively).

To investigate the effect of MQ on ESCC cell proliferation, we examined cell proliferation and anchor-independent cell growth of KYSE150 and KYSE450 cells after treatment with different doses of MQ (0, 1, 2.5, 5, and 10 μM) using a cell proliferation and anchor-independent cell growth assay. Results indicated that MQ inhibited proliferation of KYSE150 and KYSE450 cells (Figure 1A). Anchorage-independent growth of KYSE150 and KYSE450 cells was also significantly suppressed after MQ treatment, especially with 10 μM MQ (Figure 1B). Plate clone formation assays also showed smaller and reduced clones in MQ-treated groups (Figure 1C). These data suggested that MQ treatment suppresses ESCC proliferation and clone formation at the cellular level. Besides, MQ did not show obvious toxicity on SHEE cell line (Figures S1A–C).

### Mass Spectrometric Analysis of Proteomic Changes of KYSE150 Cells After MQ Treatment

**Figure 2:**
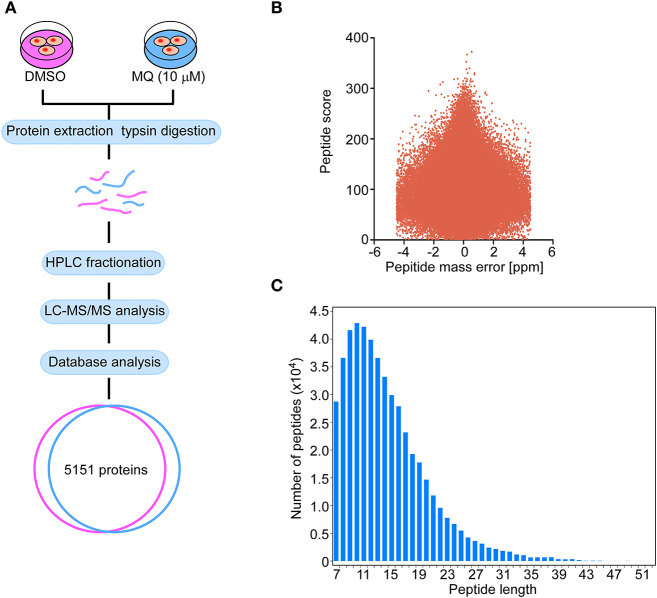
Flow graph of proteomics. **(A)** Schematic diagram of the quantitative proteomics analysis process. KYSE150 cells were treated with DMSO or MQ (10 μM) for 24 h, after LC-MS/MS analysis, 5,151 proteins were identified in the overlap section between DMSO and MQ (10 μM) groups. **(B)** Volcano diagram indicated the distribution of 5,151 proteins identified. Peptide mass error was plotted as x axis, the peptide score was plotted as y axis. **(C)** The distribution of peptide length of 5,151 proteins identified. Peptide length was plotted as x axis, the number of peptides was plotted as y axis.

Proteomics was used to further explore the underlying mechanisms of MQ treatment and to provide a global analysis of protein level changes after treatment. There were 5,151 of coverage and overlap of proteins identified by our proteomics approach. The flowchart and quality control profile for this analysis were shown in Figures 2A–C. We chose a 1.5-fold change from baseline as the threshold, with a *t*-test *p* < 0.05 as standard, and qualified protein level changes exceeding this range as differentially expressed proteins. Among these differentially expressed proteins, 86 proteins were up-regulated, and 61 proteins were down-regulated in cells treated with 10 μM MQ as compared to control.

### MQ Remodels Proteome in KYSE150 Cells

**Figure 3:**
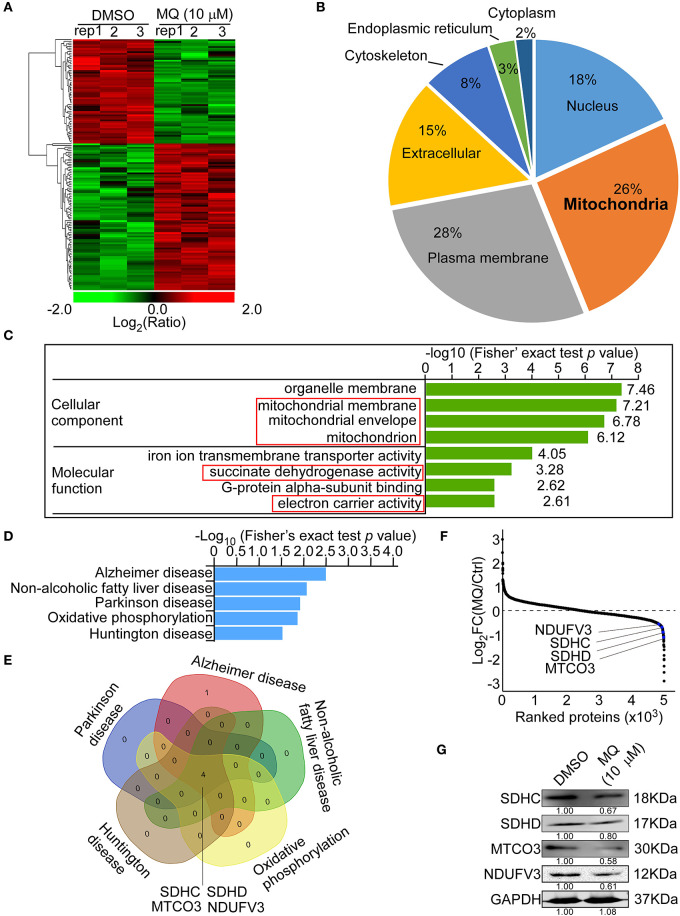
Mefloquine remodels proteome of KYSE150 cells. MS analysis of global protein changes in KYSE150 cells after MQ (10 μM) treatment for 24 h compared with DMSO treatment. **(A)** Heat map representation of differentially expressed proteins between DMSO and MQ (10 μM) groups. The color key shows the log2 transformed protein amount ratios; **(B)** WoLF PSORT software analysis of the sub-cellular localization of differentially changed proteins; **(C)** Gene ontology term enrichment classification of differentially changed proteins according to cellular component and molecular function, with the top 4 related components and functions shown; **(D)** KEGG pathway enrichment map of down-regulated pathways (blue bars). Data was shown as -Log10 (Fisher's exact test *p-*value); **(E)** Venn diagram indicating the overlap between the 5 downregulated signaling pathways. Four overlapping proteins were listed; **(F)** Averaged quantitative kinase data from the full proteome, ranked according to their Log2 FC between the DMSO and MQ-treated cells, and the locations of SDHC, SDHD, MTCO3, and NDUFV3 were shown; **(G)** Western blot for SDHC, SDHD, MTCO3, and NDUFV3 in KYSE150 cells after DMSO or MQ (10 μM) treatment.

MQ treatment caused a series of proteins to be differentially expressed in KYSE150 cells (Figure 3A). WOLF PSORT software was used to classify the sub cellular localization of the differential expression proteins, showing that 26% of the differentially expressed proteins localized to the mitochondria (Figure 3B). Although plasma membrane was more affected (28%) compared with mitochondria (26%). Gene ontology analysis also showed that the mitochondria were more sensitive to MQ treatment, based on cellular component and molecular function classification (Figure 3C). The cellular components identified as being affected by MQ treatment included the mitochondrial membrane, mitochondrial envelope, and mitochondrion, and affected molecular functions included succinate dehydrogenase activity and electron carrier activity. Meanwhile, a KEGG pathway enrichment map showed the top five signaling pathways (Figure 3D), which were downregulated after MQ treatment. Venn diagram analyses indicated that mitochondrial proteins succinate dehydrogenase complex subunit C (SDHC), succinate dehydrogenase complex subunit D (SDHD), mitochondrially encoded cytochrome c oxidase III (MTCO3), and NADH: ubiquinone oxidoreductase subunit V3 (NDUFV3) were all involved in these pathways (Figure 3E). In addition, the extensive protein reprogramming highlights a visible decrease of SDHC, SDHD, MTCO3, and NDUFV3 in MQ-treated cells (Figure 3F). A separate table contains a list of the top down differentially regulated genes affected by the treatment was also shown in Table S1. Western blot also verified that protein levels of SDHC, SDHD, MTCO3, and NDUFV3 were decreased in KYSE150 cells after MQ treatment (Figure 3G). Therefore, based on the results of sub cellular localization classification, Gene ontology analysis and KEGG pathway enrichment, mitochondria dysfunction may play a vital role in MQ-induced anti-tumor effect.

### MQ Induces Mitochondrial Autophagy of KYSE150 Cells

**Figure 4:**
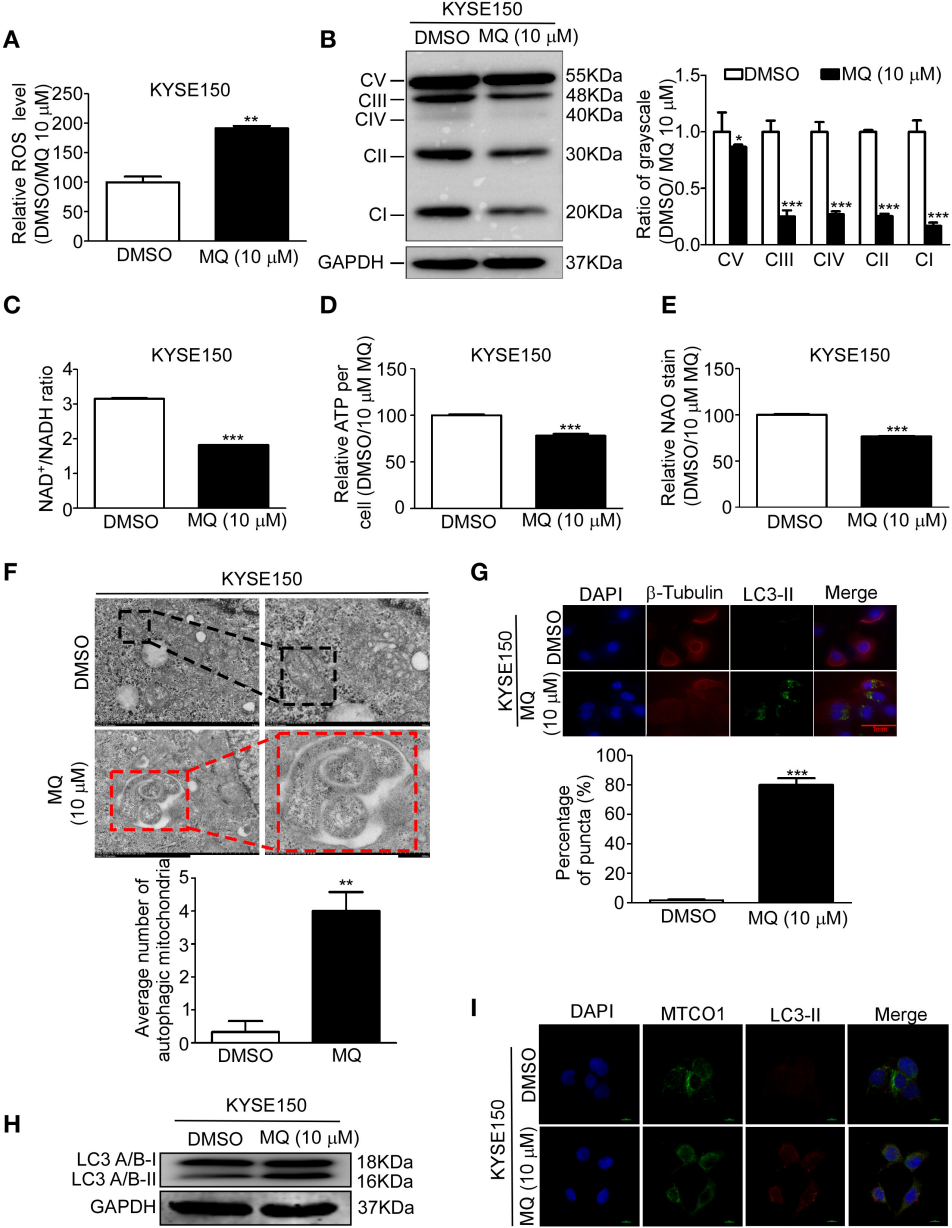
Mefloquine induces mitochondrial autophagy of KYSE150 cells. **(A)** ROS generation in KYSE150 cells after DMSO and MQ (10 μM) treatment for 24 h was measured with ROS assay kit; **(B)** KYSE150 cells probed for OXPHOS protein levels (complexes I, II, III, IV, and V) after DMSO or MQ (10 μM) treatment for 24 h; **(C)** NADH was quantified in KYSE150 cells after DMSO and MQ (10 μM) treatment for 24 h by NAD^+^/NADH assay kit; **(D)** ATP generation was tested in KYSE150 cells after DMSO and MQ (10 μM) treatment for 24 h by ATPlite assay kit; **(E)** Mitochondrial mass was evaluated with NAD staining in mitochondria after DMSO and MQ (10 μM) treatment for 24 h; **(F)** KYSE150 cells were photographed by transmission electron microscopy after DMSO or MQ (10 μM) treatment for 24 h. Three electron microscopy pictures were randomly chosen, and the number of autophagic mitochondria was counted. Black boxes indicate the normal mitochondria, and red boxes indicate the autophagosomes engulfing mitochondria. Scale = 500 nm; **(G)** LC3-II labeling for autophagic mitochondria after 24 h of DMSO or 10 μM MQ treatment by immunofluorescence analysis; **(H)** Western blot for LC3A/B in KYSE150 cells after DMSO and MQ (10 μM) treatment for 24 h; **(I)** Co-localization analysis of MTCO1 and LC3-II in KYSE150 cells after DMSO and MQ (10 μM) treatment for 24 h by immunofluorescence analysis.

Based on our proteomics results, mitochondria seemed to be particularly affected by MQ treatment. We therefore explore the specific effect of MQ on the mitochondrial function of KYSE150 and KYSE450 cells. ROS production of mammalian mitochondria plays a vital role in underlying oxidative damage in many pathologies and contributes to retrograde redox signaling from the organelle to the cytosol and nucleus (16). ROS generation increased in KYSE150 (Figure 4A) and KYSE450 (Figure S4A) cells after MQ (10 μM) treatment for 24 h. Mitochondria is best known for producing ATP via oxidative phosphorylation (OXPHOS) (17). Analysis of protein expression levels of 5 OXPHOS complexes subunits (CI subunit NDUFB8, CII-30 kDa, CIII-Core protein 2, CIV subunit I and CV alpha subunit) showed that all were downregulated after MQ treatment (Figure 4B), which suggested that MQ induced mitochondrial disorder. To further clarify mitochondrial impairment after MQ treatment, we looked at nicotinamide adenine dinucleotide, as the maintenance of an optimal NAD^+^/NADH ratio is essential for mitochondrial function (18). NAD^+^/NADH ratio decreased after MQ (10 μM) treatment in KYSE150 (Figure 4C) and KYSE450 (Figure S4B) cells, as well as ATP production in KYSE150 (Figure 4D) and KYSE450 (Figure S4C) cells. Besides, NAO was applied to evaluate mitochondrial mass in KYSE150 and KYSE450 cells after MQ (10 μM) treatment for 24 h. Mitochondrial mass decreased in KYSE150 (Figure 4E) and KYSE450 (Figure S4D) cells. Meanwhile, we found that morphology of mitochondria was also altered in MQ-treated KYSE150 cells through transmission electron microscopy, with treated cells exhibiting many double-membraned autophagic vesicles compared with the DMSO group (Figure 4F, Figure S2A). LC3 plays a vital role in autophagosome membrane biogenesis (19, 20), where LC3- I is converted into a phosphatidylethanolamine-conjugated LC3- II form when cells undergo autophagy (21). Based on the formation of autophagic vesicles, we probed for LC3-II protein levels by immunofluorescence analysis of KYSE150, LC3-II signal (green) enhanced after MQ (10 μM) treatment for 24 h in KYSE150 cells (Figure 4G). Western blot found that LC3- II protein level increased after MQ treatment in KYSE150 cells (Figure 4H). Meanwhile, co-localization of mitochondrial marker (MTCO1) and autophagy marker (LC3-II) was analyzed with immunofluorescence. Co-localization of MTCO1 and LC3-II was found in KYSE150 (Figure 4I) and KYSE450 (Figure S4E) cells after MQ (10 μM) treatment for 24 h. While the co-localization of MTCO1 and LC3-II looks like not very strong, and there are two possible reasons. One reason is that LC3-II signal is not as strong as DAPI and MTCO1; the other reason is that not all the mitochondria in cells undergo autophagy after MQ (10 μM) treatment. In addition, mitochondria as an important organelle to maintain intracellular homeostasis, apoptosis, and necrosis, we then tested the effect of MQ on cell apoptosis. KYSE150 cells did not show obvious apoptosis after MQ (10 mM) treatment at 48 and 72 h (Figure S2B). Therefore, we believe that the antitumoral effect of MQ is a consequence of the proliferation inhibition.

### SDHC Is Highly Expressed in ESCC Tissues and Knocking SDHC Down Suppresses Cell Proliferation of ESCC

**Figure 5:**
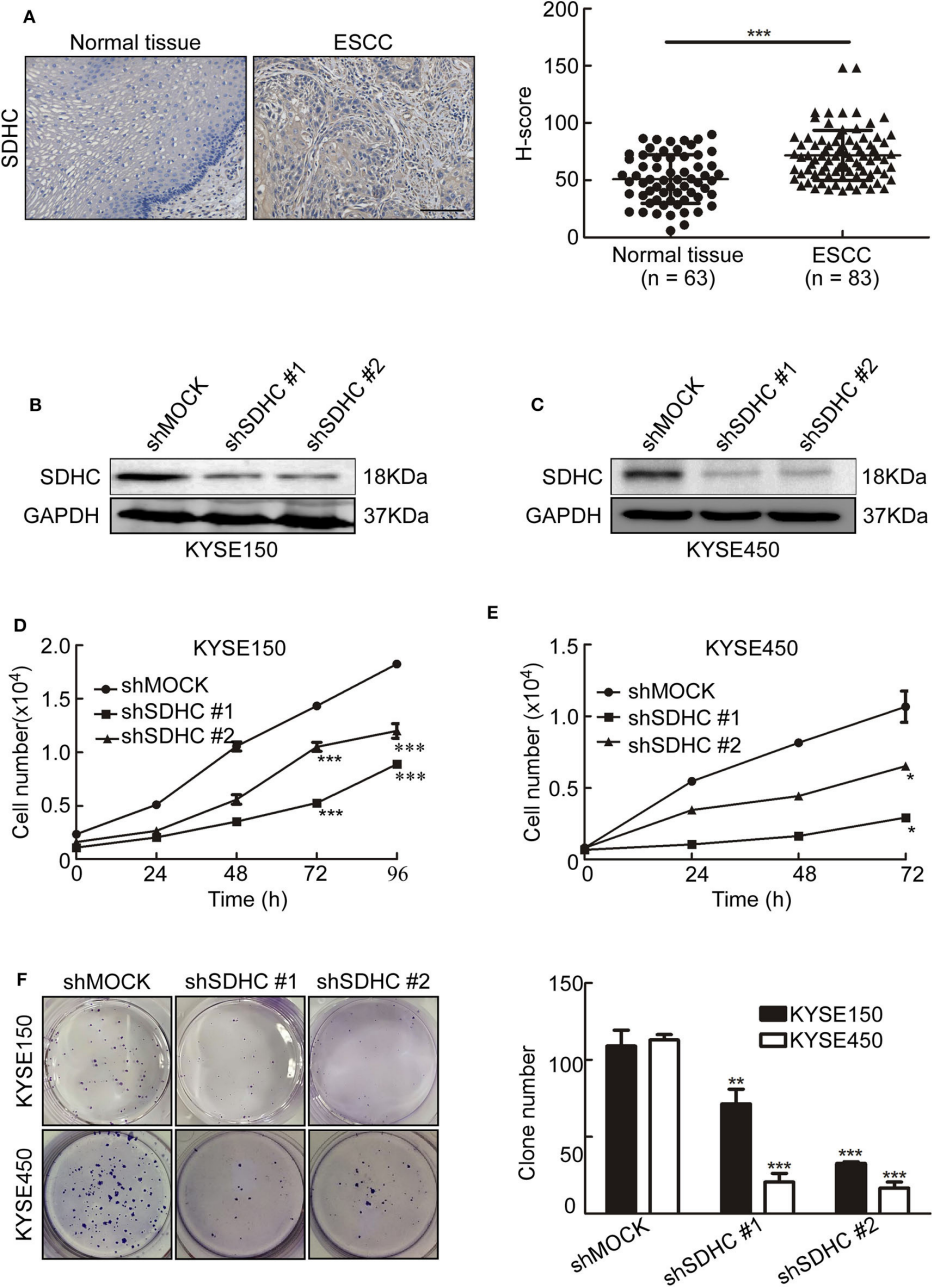
SDHC is highly expressed in ESCC tissues and knocking SDHC down suppresses proliferation of ESCC cells. **(A)** ESCC tissue spots were incubated with SDHC antibody, then stained with DAB and DAB density was analyzed for ESCC and normal tissues. The asterisks (***) indicate a significant change (*p* < 0.001); **(B)** Short hairpin RNA (sh*SDHC*) in vector plko.1 was used to transduce KYSE150 and KYSE450 cells, with knock-down efficiency tested by Western blot in KYSE150 and **(C)** KYSE450 cells; **(D)** Cell count for KYSE150 and **(E)** KYSE450 cells at 0, 24, 48, 72, and 96 h; **(F)** Colony formation for KYSE150 and KYSE450 cells after 10 days. All data are shown as means ± S.D. The asterisks (*, **, ***) indicate a significant decrease (*p* < 0.05, *p* < 0.01, *p* < 0.001, respectively).

Since MQ treatment perturbed mitochondrial activity and induced mitochondrial autophagy, we aimed to identify which down-regulated mitochondrial proteins could play a role in MQ-induced autophagy. SDHC, SDHD, MTCO3, and NDUFV3 were identified as being possible candidates. Succinate dehydrogenase as a key component of the mitochondrial respiratory chain (RC) of all living organisms (22), SDH mutation frequently occurred in cancer (23–25). SDHC has a higher mutation rate compared with SDHD (26). Firstly, we tested the SDHC protein level in human ESCC tissues and found the protein level of SDHC was higher in ESCC tissues than normal tissues (Figure 5A). To further investigate the role of SDHC in ESCC tumor growth, we transfected KYSE150 and KYSE450 cells with sh*SDHC*. The transfection efficiency was tested by Western blot in KYSE150 (Figure 5B) and KYSE450 cells (Figure 5C). Meanwhile, cell proliferation of KYSE150 (Figure 5D) and KYSE450 (Figure 5E) were significantly suppressed after knocking SDHC down. Furthermore, clone formation was also obviously inhibited (Figure 5F). This indicated that SDHC plays an important role in tumor growth.

### MQ Inhibits Tumor Growth of ESCC *in vivo*

**Figure 6:**
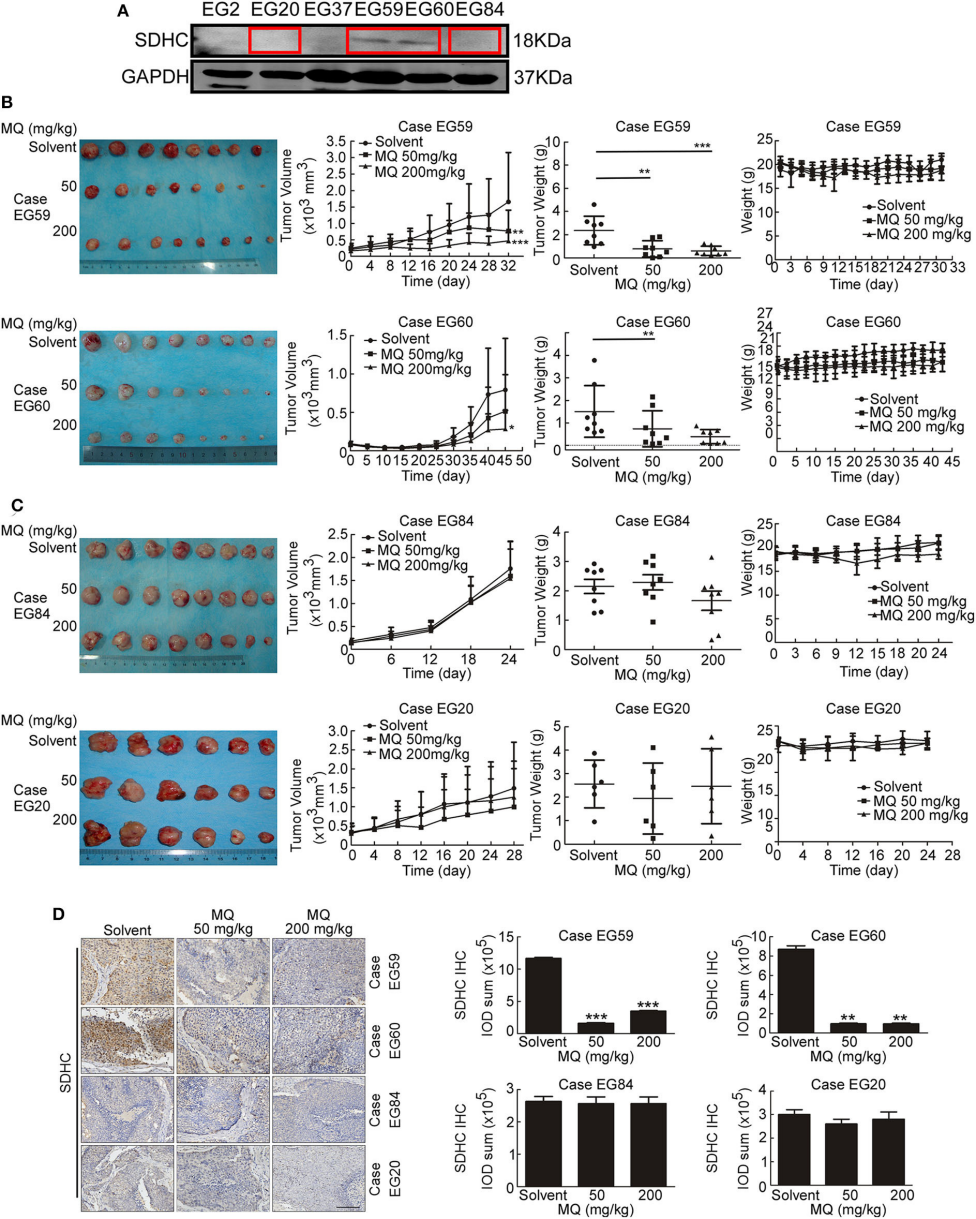
Mefloquine inhibits tumor growth of ESCC PDX models. **(A)** SDHC protein level was detected in cases of ESCC tissues by Western blot; **(B,C)** Effect of MQ treatment on tumor growth. MQ significantly suppressed tumor growth of case EG59 and EG60, while not in case EG20 and EG84; **(D)** Immunohistochemistry analysis of SDHC with DAB staining in case EG59, EG60, EG20, and EG84 after MQ treatment. All data are shown as means ± S.D. The asterisks (*, **, ***) indicate a significant decrease (*p* < 0.05, *p* < 0.01, *p* < 0.001, respectively).

To investigate whether the anti-tumor effect of MQ are SDHC dependent or not *in vivo*, we established PDX mouse models of ESCC (15). 4 cases of ESCC PDXs were chosen for experiment, including case EG59, case EG60, case EG20, and case EG84. The protein levels of SDHC are high in case EG59 and EG60, while the protein levels are low in case EG20 and EG84 (Figure 6A), while not the same with SDHD, MTCO3 and NDUFV3 (Figure S3). Mice were treated with solvent (0.9% normal saline) or MQ (50 or 200 mg/kg) by oral gavage, once a day. Interestingly, MQ clearly suppressed tumor growth in case EG59 (Figure 6B, upper) and EG60 (Figure 6B, lower), while MQ did not exhibit any distinct inhibitory effect in case EG84 (Figure 6C, upper) and EG20 (Figure 6C, lower). Moreover, tumor weights also clearly decreased in case EG59 and EG60 (Figure 6B) after MQ treatment, while not in case EG84 and EG20 (Figure 6C). There was almost no difference of body weights amongst the 3 treatment groups in these 4 cases (Figures 6B,C). Furthermore, IHC analyses demonstrated that SDHC protein levels were also decreased in MQ treated groups of EG59 and EG60 compared to solvent control. However, there was no evident difference between solvent control and MQ-treated group of EG84 and EG20 (Figure 6D). These results suggest that SDHC may play a vital role in MQ-induced anti-tumor effect of ESCC.

## Discussion

With the current treatment, the 5-year survival rate of ESCC is still low worldwide (27, 28), especially in developing countries. One major reason is lacking recurrence prevention drugs (29, 30). According to retrospective studies, many drugs marketed for other diseases have shown an anti-tumor effect, which make them probably can be used for cancer recurrence prevention (31–33). Due to time-saving, lower cost, and streamlined approval processes, screening FDA-approved non-anti-tumor drugs for cancer prevention or recurrence prevention is attractive (34, 35). Through such a strategy, we found that MQ, released as an anti-malarial drug, suppressed the proliferation and clone formation of ESCC cells *ex vivo*, which was consistent with reports that MQ played a vital role in inhibiting tumor cell proliferation in other cancer types (9, 10, 36).

Mitochondrial autophagy has been reported to play a vital role in human diseases, including cancer (37). However, the relationship between autophagy and cancer is still obscure (38, 39). The role of autophagy in cancer may depend on the environment and the stage of tumorigenesis (40). In our study, global proteome analysis disclosed a more detailed picture of the protein changes in MQ-treated KYSE150 cells compared with control cells, and we found that MQ could induce mitochondrial autophagy in KYSE150 cells. Although it has been reported that MQ suppressed cancer cells proliferation (41, 42), our experiment firstly profiled the global proteome and validated that MQ mainly affected mitochondria and induced autophagy in ESCC cells.

Succinate dehydrogenase is an important tricarboxylic-acid-cycle-related enzyme in the mitochondria, and mutations to this enzyme frequently occur in cancer (43, 44). It has also been reported that the mutation rate of SDHC was higher among the succinate dehydrogenase complex subunits in cancer (26). Therefore, when we showed that SDHC was downregulated in MQ-treated ESCC cells, we hypothesized that SDHC may play an important role in ESCC tumor growth. Indeed, we found that SDHC protein level was higher expressed in ESCC tissues compared with normal tissues (Figure 5A). Moreover, knocking down SDHC inhibited cell proliferation and clone formation in KYSE150 and KYSE450 cells (Figures 5B–F). Based on the *ex vivo* studies, a PDX mouse model was used to explore the anti-tumor effect of MQ *in vivo*. Interestingly, MQ significantly suppressed tumor growth of PDXs with high level of SDHC, while not having the same effect in PDXs with low level of SDHC. These data indicated that SDHC probably plays an important role in MQ-induced mitochondrial autophagy. Further studies about the relationship of SDHC, SDHD, MTCO3, and NDUFV3 in MQ-induced anti-tumor effect will be applied. It's reported that mefloquine has been seen to cause serious neuropsychiatric adverse effects on rare occasions (45). In our study, mice with xenografts from case EG59 were a little irritability in mental state after MQ administration, while this was not the case for the other xenograft groups. Further evaluation is required to determine if this is an effect of MQ or an outlying event.

## Conclusions

Collectively, this study showed that MQ treatment induced mitochondrial autophagy and suppressed tumor growth of ESCC. Moreover, SDHC may play an important role in the MQ-induced anti-tumor effect of ESCC.

## Standard Biosecurity and Institutional Safety Procedures Statement

We adhered to standard biosecurity and institutional safety procedures of Zhengzhou University.

## Data Availability Statement

The raw data supporting the conclusions of this article will be made available by the authors, without undue reservation, to any qualified researcher.

## Ethics Statement

This study was approved by the Research Ethics Committee of Zhengzhou University. Written informed consent was provided by all patients for the use of the tissue samples.

## Author Contributions

KL: conceptualization. YX, JZhan, JZhao, and QY: data curation. YX, JZhan, and XC: formal analysis. YX, JZhan, XL, YW, QY, XZ, BLu, BLi, and ZB: methodology. KL and ZD: supervision. YX: writing—original draft. All authors contributed to the article and approved the submitted version.

## Conflict of Interest

The authors declare that the research was conducted in the absence of any commercial or financial relationships that could be construed as a potential conflict of interest.
