# Supplementary material for: Mefloquine Inhibits Esophageal Squamous Cell Carcinoma Tumor Growth by Inducing Mitochondrial Autophagy
Source: Front Oncol. 2020 Jul 28;10:1217. doi: 10.3389/fonc.2020.01217 (PMC7400730; doi:10.3389/fonc.2020.01217)
Supplement: Supplementary file 1 [file Table_1.pdf]

Protein accession	Protein description	MQ/Ctr Ratio	Regulated Type	Quant category	MQ/Ctr P value	Gene name	Sequence coverage [%]	Mol. weight [kDa]	Score
O15235	28S ribosom	0.666	Down	Q1	0.0140247	MRPS12	33.3	15.173	26.866
O14817	Tetraspanin	0.665	Down	Q1	0.0109632	TSPAN4	16	26.118	8.0014
Q99519	Sialidase-1	0.665	Down	Q1	0.0017567	NEU1	14.9	45.467	11.095
Q9BQE4	Selenoprote	0.664	Down	Q1	0.0158967	VIMP	36.5	21.163	72.372
Q9BU23	Lipase matu	0.663	Down	Q1	0.035378	LMF2	10.2	79.697	17.473
P51636	Caveolin-2	0.662	Down	Q1	0.000837	CAV2	52.5	18.291	43.973
O43819	Protein SCO	0.66	Down	Q1	0.02538	SCO2	25.2	29.81	29.444
Q15043	Zinc transpo	0.659	Down	Q1	0.000121	SLC39A14	5.1	54.212	5.0869
P24390	ER lumen p	0.655	Down	Q1	0.034203	KDELR1	25.9	24.542	5.8426
P56181	NADH dehy	0.654	Down	Q1	0.03726	NDUFV3	14.8	11.94	6.8834
Q5BJD5	Transmemb	0.653	Down	Q1	0.000223	TMEM41B	11.7	32.513	65.237
O75414	Nucleoside	0.652	Down	Q1	0.032304	NME6	24.2	21.142	8.5702
P02786	Transferrin	0.65	Down	Q1	3.982E-05	TFRC	49.2	84.87	231.07
Q9Y5W7	Sorting nexi	0.648	Down	Q1	0.02986	SNX14	12.5	110.18	37.266
Q53S33	BolA-like p	0.644	Down	Q1	0.024782	BOLA3	26.2	12.114	14.66
Q658P3	Metalloredu	0.644	Down	Q1	0.029679	STEAP3	15.4	54.6	52.995
Q99470	Stromal cell	0.644	Down	Q1	0.0061609	SDF2	36	23.026	87.598
O95140	Mitofusin-2	0.642	Down	Q1	0.0170578	MFN2	32.2	86.401	75.368
Q99643	Succinate de	0.64	Down	Q1	0.022082	SDHC	12.4	18.61	4.0603
Q96EL2	28S ribosom	0.638	Down	Q1	0.0067952	MRPS24	40.1	19.015	33.238
Q8WVQ1	Soluble calc	0.638	Down	Q1	0.0008442	CANT1	40.1	44.839	40.6
Q9Y3Q3	Transmemb	0.637	Down	Q1	0.0029843	TMED3	18.9	24.777	13.898
O15460	Prolyl 4-hyd	0.632	Down	Q1	0.0101986	P4HA2	30.5	60.901	66.257
Q9H254	Spectrin bet	0.631	Down	Q1	0.021797	SPTBN4	1.7	288.98	1.4014
P33121	Long-chain-	0.63	Down	Q1	0.0001611	ACSL1	19.3	77.942	16.412
P0DJ07	Protein PET	0.629	Down	Q1	0.0008575	PET100	13.7	9.1135	4.212
Q92934	Bcl2-associa	0.628	Down	Q1	0.0049753	BAD	50.6	18.392	29.238
Q9NWU5	39S ribosom	0.625	Down	Q1	3.593E-05	MRPL22	43.7	23.64	35.162
Q5T9L3	Protein wntl	0.624	Down	Q1	0.0003791	WLS	10.9	62.253	31.585
Q13829	BTB/POZ d	0.624	Down	Q1	0.0016796	TNFAIP1	24.7	36.204	11.024
O14521	Succinate de	0.622	Down	Q1	1.934E-05	SDHD	6.3	17.043	4.5741
Q9UPV0	Centrosoma	0.591	Down	Q1	5.648E-05	CEP164	1.7	164.31	3.716
Q9BT17	Mitochondr	0.585	Down	Q1	0.0118608	MTG1	12.9	37.236	20.044
Q5T653	39S ribosom	0.576	Down	Q1	0.0058973	MRPL2	34.4	33.3	79.291
Q7Z7F7	39S ribosom	0.575	Down	Q1	0.0018235	MRPL55	31.2	15.128	10.009
Q92520	Protein FAM	0.574	Down	Q1	0.0006834	FAM3C	64.3	24.68	74.524
Q9NYZ1	Golgi appar	0.574	Down	Q1	0.0003621	TVP23B	14.6	23.576	6.1174
Q86WB0	Nuclear-inte	0.561	Down	Q1	0.0012989	ZC3HC1	24.5	55.261	18.277
Q15776	Zinc finger	0.554	Down	Q1	0.005903	ZKSCAN8	12.6	65.815	23.883
Q9NX00	Transmemb	0.552	Down	Q1	0.032984	TMEM160	14.9	19.657	5.3955
O95563	Mitochondr	0.546	Down	Q1	0.0091012	MPC2	18.9	14.279	7.4576
Q9H7H0	Methyltrans	0.532	Down	Q1	0.0074021	METTL17	9.4	50.733	13.163
Q8NBJ4	Golgi memb	0.498	Down	Q1	2.087E-05	GOLM1	36.2	45.333	108.41
P62491	Ras-related	0.485	Down	Q1	0.0067405	RAB11A	63.4	24.393	12.151

P00414	Cytochrome	0.482	Down	Q1	0.000936	MT-CO3	8.8	29.95	34.995
P36639	7,8-dihydro-	0.459	Down	Q1	0.0011037	NUDT1	14.2	22.519	17.286
Q15392	Delta(24)-st	0.415	Down	Q1	0.0001554	DHCR24	22.5	60.101	14.096
Q9Y320	Thioredoxin	0.362	Down	Q1	7.934E-05	TMX2	41.9	34.037	31.61
P55061	Bax inhibito	0.285	Down	Q1	0.020436	TMBIM6	9.3	26.537	4.7298

MS/MS Counts	Peptides	Unique peptides	MS/MS Count rep1_Ctrl	MS/MS Count rep1_MQ	MS/MS Count rep2_Ctrl
23	4	4	5	3	4
14	3	3	2	2	2
23	6	6	5	2	3
26	6	6	2	6	5
15	5	5	3	3	3
45	5	5	6	9	8
18	5	5	3	2	4
20	2	2	3	2	3
12	4	4	2	2	2
13	1	1	2	2	2
29	3	3	4	5	5
9	4	4	3	1	3
285	32	32	54	45	50
18	9	9	3	3	7
14	2	2	4	2	3
18	5	5	3	3	3
29	5	5	5	5	4
94	23	23	19	14	20
20	2	2	3	3	4
33	5	5	6	6	6
50	10	10	8	7	11
12	3	3	2	2	1
64	13	13	9	9	12
7	5	2	2	0	1
41	14	14	9	5	9
10	2	2	2	2	1
26	5	5	4	3	5
48	9	9	9	6	8
33	5	5	6	5	7
23	7	5	4	4	3
11	1	1	2	2	2
14	3	3	2	2	3
27	4	4	6	5	5
35	8	8	7	7	7
8	3	3	3	0	2
99	14	14	20	13	18
21	3	3	4	2	4
36	9	9	8	4	8
18	6	5	2	3	3
13	3	3	2	3	2
12	3	3	3	1	3
18	4	4	6	3	4
62	14	14	12	7	12
23	14	2	5	3	5

14	2	2	3	1	2
19	2	2	3	3	3
32	11	11	7	4	9
41	11	11	11	5	10
10	2	2	4	0	2
