# Supplementary material for: Mefloquine Inhibits Esophageal Squamous Cell Carcinoma Tumor Growth by Inducing Mitochondrial Autophagy
Source: Front Oncol. 2020 Jul 28;10:1217. doi: 10.3389/fonc.2020.01217 (PMC7400730; doi:10.3389/fonc.2020.01217)
Supplement: Supplementary file 2 [file Data_Sheet_1.DOCX]

**Supplementary Figure Legends**

**Figure S1. MQ did not show obvious toxicity in Shantou human embryonic esophageal (SHEE) cell line. (A)** SHEE cells were treated with MQ (0, 1, 2.5, 5 and 10 μM) for 24 h. Cell viability was measured by cell count. The n.s. indicated that there is no significant difference between MQ-treated group and control group; **(B)** Mitochondria morphology of SHEE cells after DMSO and MQ (10 μM) treatment was observed by transmission electron microscopy. The black boxes indicate the normal mitochondria; **(C)** The protein expression levels of SDHC, SDHD, MTCO3 and NDUFV3 in SHEE cells after DMSO and MQ (10 μM) treatment were also measured by Western blot.

**Figure S2. MQ induced mitochondrial autophagy in KYSE150 cells, while not induce apoptosis of KYSE150 cells.** **(A)** KYSE150 cells were photographed by transmission electron microscopy after MQ (10 μM) treatment for 24 h. The red boxes indicate the autophagosomes engulﬁng mitochondria; **(B)** KYSE150 cells were treated with DMSO and MQ (10 μM) for 48 h and 72 h. Cells were stained with PI and Annexin V and analyzed by flow cytometer.

**Figure S3. SDHC was high expressed in case EG59 and EG60, low expressed in case EG20 and EG84, while not the same with SDHD, MTCO3 and NDUFV3. (A)** The protein expression levels of SDHC, SDHD, MTCO3 and NDUFV3 were measured in several PDX cases by Western blot.

**Figure S4. MQ impaired mitochondria function of KYSE450 cells. (A)** ROS generation in KYSE450 cells after DMSO and MQ (10 μM) treatment for 24 h was measured with ROS assay kit; **(B)** NAD+ and NADH were quantified in KYSE450 cells after DMSO and MQ (10 μM) treatment for 24 h by NAD+/NADH assay kit; **(C)** ATP generation was tested in KYSE450 cells after DMSO and MQ (10 μM) treatment for 24 h by ATPlite assay kit; **(D)** Mitochondrial mass in KYSE450 cells was evaluated with NAD staining in mitochondria after DMSO and MQ (10 μM) treatment for 24 h; **(E)** Co-localization analysis of MTCO1 and LC3-II in KYSE450 cells after DMSO and MQ (10 μM) treatment for 24 h by immunofluorescence analysis.
