# Supplementary figures and images for: Mefloquine Inhibits Esophageal Squamous Cell Carcinoma Tumor Growth by Inducing Mitochondrial Autophagy
Source: Front Oncol. 2020 Jul 28;10:1217. doi: 10.3389/fonc.2020.01217 (PMC7400730; doi:10.3389/fonc.2020.01217)

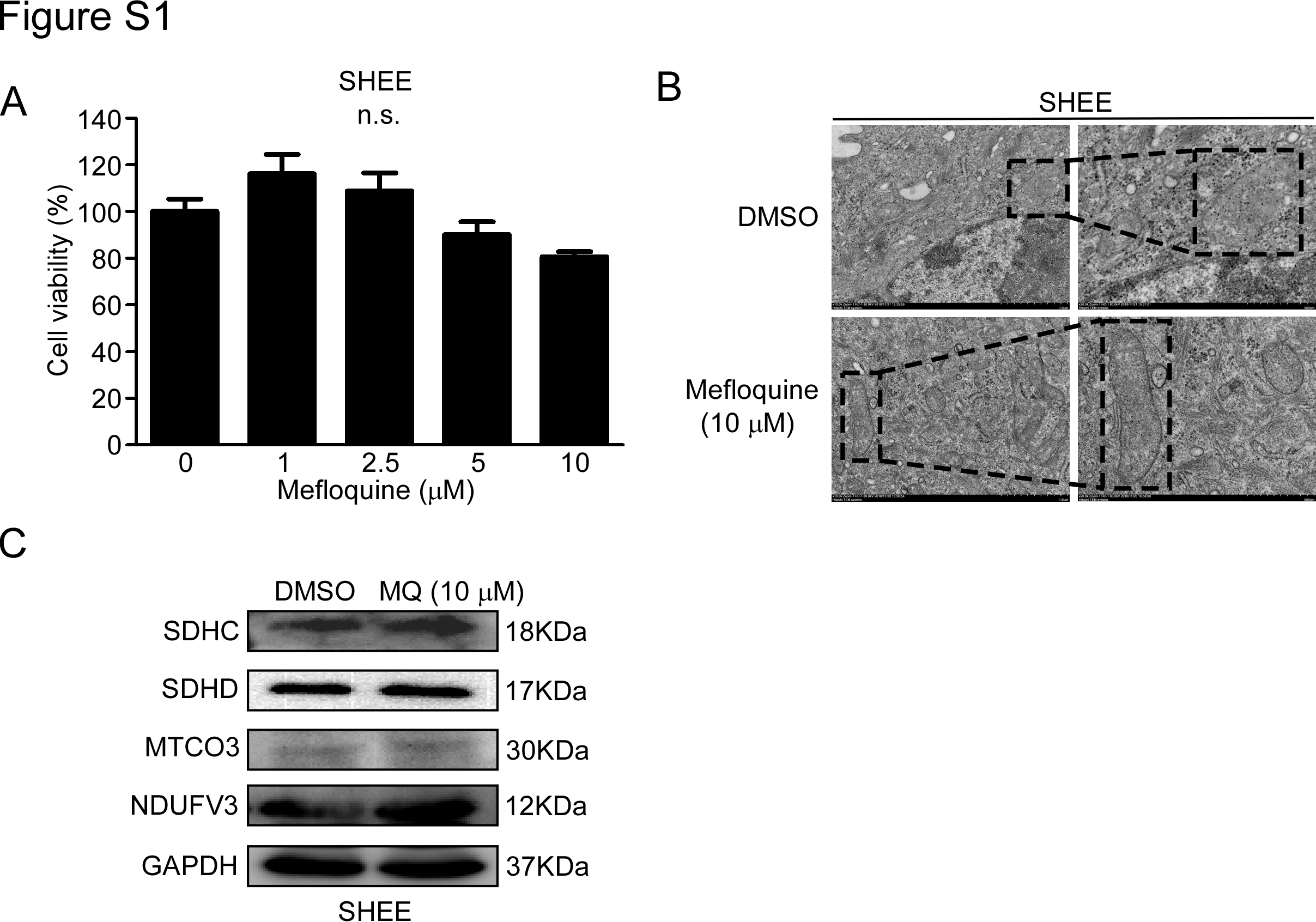

Supplement: Supplementary file 3 [file Image_1.tif]

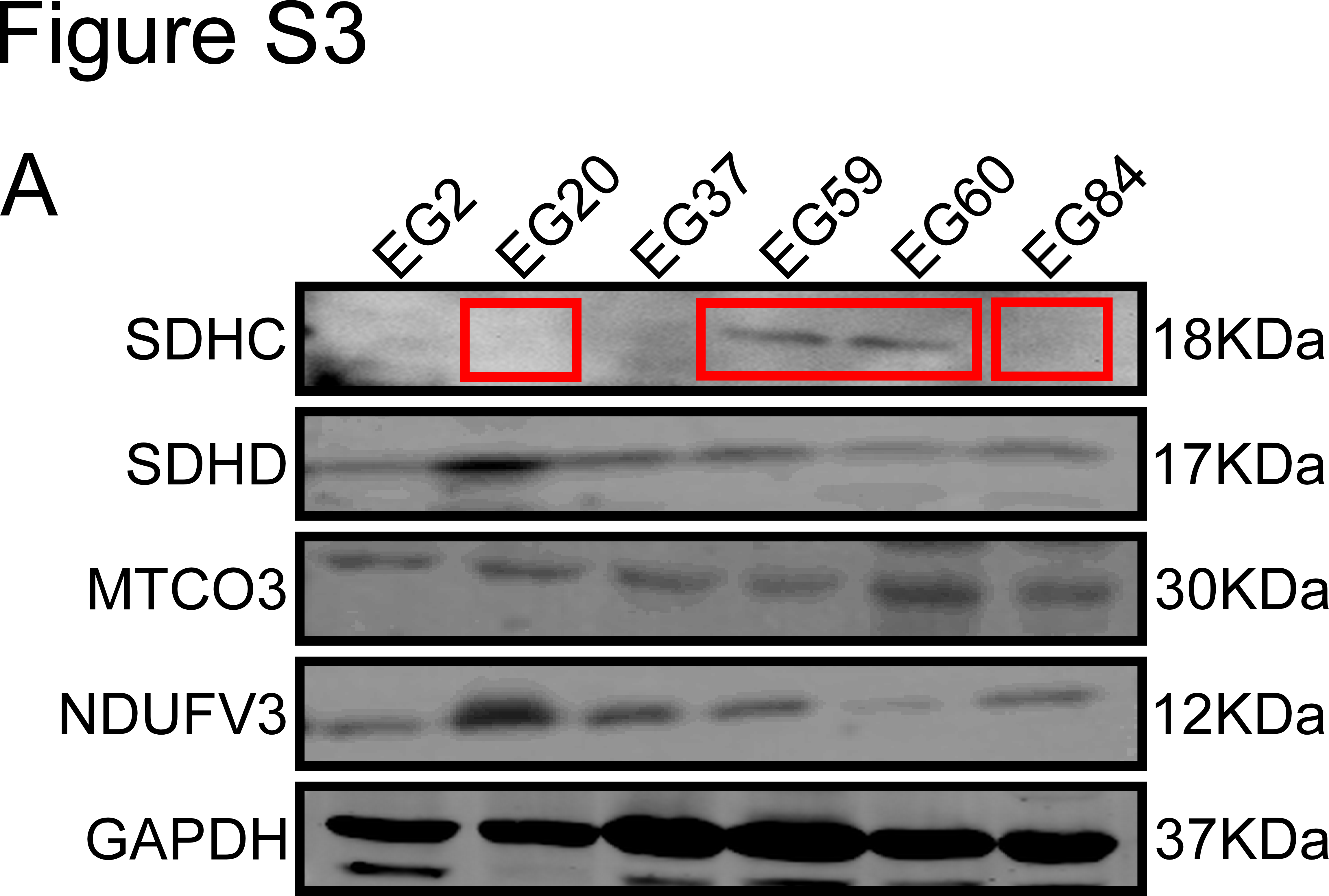

Supplement: Supplementary file 5 [file Image_3.TIF]

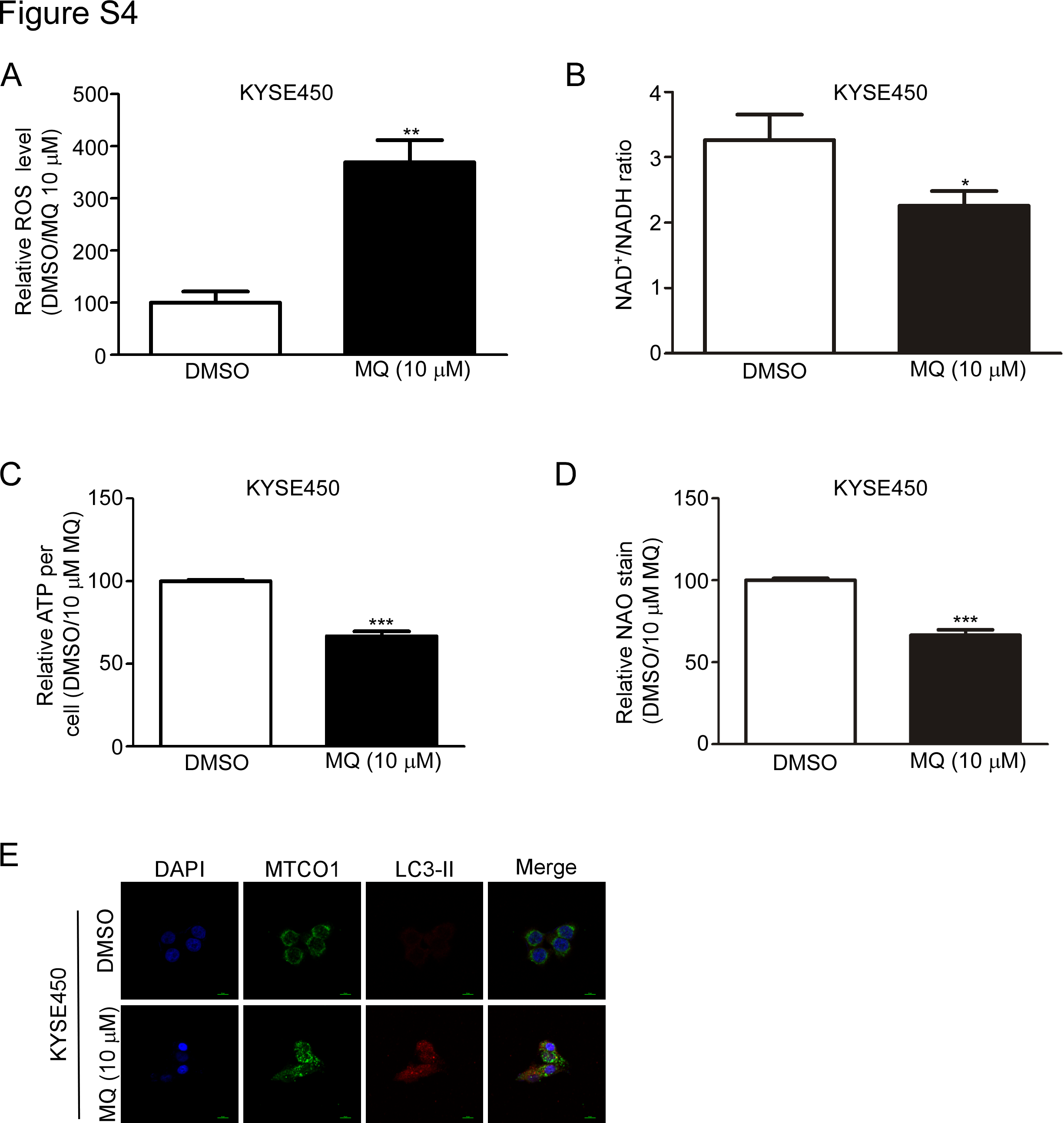

Supplement: Supplementary file 6 [file Image_4.tif]
